# Relapse to problem drinking or trading up to spirits? Using U.S. national cross-sectional survey data to highlight possible negative impacts of potential tobacco retail changes

**DOI:** 10.1186/s13011-022-00498-8

**Published:** 2022-11-01

**Authors:** Katherine J. Karriker-Jaffe, Lisa Henriksen, Elizabeth A. Smith, Patricia A. McDaniel, Ruth E. Malone, William C. Kerr

**Affiliations:** 1 Center on Behavioral Health Epidemiology, Implementation & Evaluation Research, RTI International, 2150 Shattuck Avenue, Suite 800, 94704 Berkeley, CA USA; 2 Stanford Prevention Research Center, Department of Medicine, Stanford University School of Medicine, 94305 Stanford, CA USA; 3 Department of Social & Behavioral Sciences, School of Nursing, University of California, San Francisco, 94143 San Francisco, CA USA; 4 Alcohol Research Group, Public Health Institute, 94608 Emeryville, CA USA

**Keywords:** Tobacco, Retail sales restrictions, Alcohol use disorder, Government monopoly systems

## Abstract

**Background:**

According to the National Alcohol Beverage Control Association, twelve states in the United States (U.S.) have government retail monopolies on spirits/liquor sales. With a new federal minimum legal sales age for tobacco (raised from 18 to 21, the minimum legal sales age for alcohol), we examine possible unintended consequences of a hypothetical policy change restricting retail tobacco sales to state-run spirits/liquor stores in alcohol control states, which has been proposed as a tobacco endgame strategy.

**Methods:**

We used cross-sectional survey data from 14,821 randomly-selected adults ages 21 and older who responded to the 2015 or 2020 U.S. National Alcohol Survey (51.8% female; 65.8% identified as non-Hispanic White, 12.4% as Black or African American, 14.2% as Hispanic or Latinx; 34.0% had a low level of education), including 2,274 respondents (18.9%) residing in one of the alcohol control states (representing 42.2 million (M) adults ages 21+). We estimated associations between tobacco measures (lifetime smoking status, lifetime daily smoking, past-year daily smoking) and alcohol measures (drinking status, beverage choices, lifetime alcohol use disorder (AUD) status, recovery status) overall and for specific subgroups.

**Results:**

In control states, 55.1% of people who smoked daily in the past year also reported lifetime AUD, including an estimated 3.56 M adults ages 21 + who reported prior (but not current) AUD. The association of daily smoking with lifetime AUD was stronger among those with low education compared to those with higher education. Further, 58.8% of people in recovery from an alcohol and/or drug problem (1.49 M adults ages 21+) smoked daily, and this was more marked among women than men in control states.

**Conclusion:**

There could be negative consequences of an endgame strategy to restructure tobacco retail sales, including increased risk for relapse to drinking among people who smoke daily, especially among women and people with low levels of education. Strategies to mitigate unintended harms would be needed if such a policy were implemented.

## Background

Policies regulating alcohol, tobacco and other drugs are rarely coherent, integrated or proportional to the potential degree of harm [1]. Coherent policies would be consistent across different dimensions, including motivation for such policies, such as a desire to protect public health vs. a desire to generate revenue. Coherent policies also would be consistent across substance types, ideally resulting in proportional policies that regulate based on level of risk associated with use of each substance. This is not currently the case in the United States (U.S.) or many other countries, where tobacco and alcohol typically are not regulated by health agencies or taxed at levels commensurate with resulting harms [2–4]. One approach to increasing policy coherence would be to bring tobacco sales under the auspices of government retail control [5–7], similar to the approach taken to regulating alcohol in twelve U.S. states. This would represent a substantial shift in U.S. tobacco control policy, which to date has included a patchwork of legislation across a wide variety of jurisdictions, with some areas experiencing very few limitations on cigarette sales. For example, of the twelve alcohol control states in the U.S., two currently require no retail license to sell cigarettes (North Carolina and Virginia) [8].

In the U.S., a major change in federal legislation in 2019 raised the minimum legal sales age for all tobacco products, including electronic nicotine delivery systems (e-cigarettes), from 18 to 21 years of age (Tobacco 21), suggesting movement toward policy coherence based on protecting certain groups (i.e., younger people) [9, 10]. At this point, there may be crucial momentum for assessing benefits – and potential drawbacks – of a hypothetical change in tobacco policy to protect public health: moving tobacco retail sales under a government control system. Under such a scenario, those twelve U.S. states with government retail control of spirits sales (that is, states where liquor is only sold in stores owned or contracted by a state government agency such as an Alcoholic Beverage Commission) would change access to tobacco products such that sales are allowed to occur only in these same state-controlled retail stores.

Arguments for and against such a policy innovation were put forth by Smith and colleagues [1], who highlighted key potential benefits of such a policy shift, including markedly reducing tobacco availability. All U.S. states that have government retail monopolies on spirits/liquor sales (including those with government-owned retail stores, such as Pennsylvania and North Carolina, as well as those with hybrid systems involving both government-owned retail stores and a limited number of contract or private retail stores, such as Alabama and Utah) would experience a drop in tobacco retail density under such a scenario. Of these states, West Virginia would see the most dramatic reduction, from 241.8 tobacco outlets/100,000 population to just 9.6 spirits outlets/100,000 population [1]. As with alcohol [11], greater tobacco availability is associated with increased use among adults [12, 13] and greater odds of initiation among youth [14–17].

One concern not discussed in depth by Smith and colleagues [1] is possible unintended consequences of selling combustible tobacco exclusively in government-controlled liquor stores. Visual cues, such as seeing someone smoking or drinking alcohol, are related to cravings of addicting substances, including both alcohol and tobacco [18, 19]. Cues also include the products themselves, as well as associated advertising or images depicting products or use (see, for example [18], for a study of advertising-induced craving in a sample of people with AUD). Given increased exposure of people who smoke to alcohol-related cues in this proposed changed retail environment, it is possible that people who smoke who also used to have alcohol problems may relapse to drinking if they can only buy tobacco in stores where the only other product for sale is alcohol, particularly spirits or liquor. Similarly, people who used to smoke who go to a state store to purchase spirits beverages may be at an increased risk of relapsing to smoking if tobacco is readily available in those retail settings.

There is a strong relationship between alcohol and tobacco use, with people who drink heavily and people with alcohol use disorder (AUD) being most likely to smoke daily and to have nicotine dependence [20]. There also is evidence of biological mechanisms contributing to co-use of alcohol and nicotine [21, 22], and studies show that quitting use of one substance can increase the likelihood of quitting the other [23, 24]. Further, in addition to substance-specific cue reactivity contributing to increased craving, evidence of cross-cue reactivity suggests that under this hypothetical change in tobacco policy, customers may be cued or prompted to buy tobacco when they originally intended to only buy alcohol, or the reverse. In a systematic review of 37 studies, most concluded that exposure to alcohol cues increased tobacco cravings, including some evidence of effects on ad libitum smoking [25]. Beneficial cross-over effects of tobacco control policies on alcohol use are also evident. For example, there is evidence of higher likelihood of AUD remission for people in states that require smoke-free bars compared to states without such laws [26].

Under current regulatory mechanisms, not all tobacco retailers are permitted to sell alcohol, and not all alcohol retailers are allowed to sell tobacco, especially in alcohol control states [27]. For example, in North Carolina (a control state), one study estimated there were over 7,200 tobacco retail outlets, 4,800 alcohol retail outlets (including state liquor stores), and 4,600 additional retail outlets that sold both tobacco and alcohol [28]. Although alcohol retail stores account for approximately 10% of tobacco retail stores nationally [27], most of the control states do not allow sales of tobacco products in state liquor stores, and they also prohibit spirits/liquor sales in gas stations, convenience stores and grocery stores—retail contexts where tobacco products are commonly sold [27]. (Exceptions are Maine and Vermont, where there are some state-contracted convenience stores licensed to sell spirits.) As such, restructuring tobacco sales to occur in state liquor stores would create a new paired retail context for strong spirits and tobacco products in particular. Thus, another concern is that people who use tobacco who normally might buy cigarettes and beer, which is more widely available at private retailers (e.g., convenience stores and grocery stores) in control states, would instead buy beverages with higher alcohol by volume (ABV) if they could only buy tobacco in state liquor stores. Further, among US adults who smoke cigarettes, 28.8% reported purchasing by the carton in 2020 [29]. The large proportion of adults who smoke cigarettes and who also drink alcohol (43.5% of adults who smoked in 2016) [30] may be similarly motivated by cost-savings of buying alcohol in large volumes and in higher ABV products.

Focusing on a hypothetical policy to restrict tobacco sales to state-run alcohol stores, we examine potential unintended consequences for both tobacco and alcohol use by conducting secondary descriptive analyses of cross-sectional national survey data from the U.S. Specifically, we ask: [1] What proportion of people who smoke daily may be at risk of relapse to problem drinking if they could only purchase tobacco in state-operated or state-contracted liquor stores? [2] What proportion of people who smoke daily and who also drink alcohol would be at risk of purchasing higher ABV beverages if they could only purchase tobacco in state liquor stores? [3] Are these patterns different for high-priority subgroups? We focus on gender, racial or ethnic, and educational differences (separately), as prior work has noted variation in co-use of alcohol and tobacco across demographic groups [20].

## Methods

### Dataset and analysis sample

We used a combined dataset from the nationally-representative 2015 and 2020 U.S. National Alcohol Surveys (NAS) [31, 32]. The 2015 NAS (N = 7,071) used computer-assisted telephone interviewing (CATI) with a list-assisted, random digit dial (RDD) sample of landline and cellular phones. The 2020 NAS (N = 9,668) included a CATI sample (n = 1,572) selected using list-assisted RDD sampling of cellular phones and a web sample recruited using two methods: a population-representative sample recruited through address-based sampling (ABS; n = 5,661) and a non-probability sample recruited from an existing web panel (n = 2,435). All interviews and surveys were conducted in English or Spanish, and both surveys oversampled Black and Hispanic respondents.

We used a subsample of cases ages 21 and older who had data on both alcohol and tobacco use (N = 14,821). This included 2,274 respondents who lived in an alcohol control state as identified by the National Alcohol Beverage Control Association (18.9% of weighted sample): Alabama, Idaho, Maine, Montana, North Carolina, New Hampshire, Oregon, Pennsylvania, Utah, Virginia, Vermont, and West Virginia. The weighted sample was 51.8% female; 65.8% of respondents were White, 12.4% were Black, 14.2% were Hispanic, and 7.6% were another race or ethnicity; and 34.0% had a low level of education.

### Measures

**Smoking measures** were ***lifetime smoking status*** (never smoked, used to smoke, currently smokes), ***daily smoking/tobacco use in the past year*** (not including e-cigarettes) because people who use tobacco more frequently would ostensibly be purchasing tobacco more often, and ***lifetime daily smoking status*** (never smoked daily, which includes some people who currently smoke occasionally as well as some people who never smoked; people who used to smoke daily; and people who currently smoke daily).

**Alcohol measures** were ***drinking status*** (lifetime abstainer, used to drink, currently drinks), ***beverage choice*** (three non-exclusive indicators for drinking beer, wine, and spirits/liquor in the past year, and another indicator for drinking spirits at least monthly in the past year), ***lifetime AUD status*** (based on reporting symptoms in 2 or more of 11 domains defined by DSM-5 [33] in the past year/current AUD, prior to the past year/former AUD, or never), and ***recovery status*** (reported being “in recovery, or used to have an alcohol or drug problem but don’t now”).

Key **demographic variables** were self-reported ***gender/sex*** (dichotomized as male or female, because very few people reported another gender identity [n = 60 in 2020 NAS; not asked in 2015]), ***race or ethnicity*** (categorized as Caucasian or White, Black or African American, Hispanic or Latinx, and Other), and ***level of education*** (dichotomized as a high school diploma or less vs. at least some college or university). Models also adjusted for respondent’s ***age*** (continuous) and ***survey wave*** (2015 vs. 2020).

### Analyses

Descriptive analyses included bivariate design-adjusted F-tests and unadjusted logistic regression models first comparing associations of smoking and alcohol use patterns and problems for respondents in government retail control states (18.9% of weighted sample) with respondents from other states, and then limiting analyses to respondents in control states (n = 2,274, representing 42.2 million adults) to estimate associations between tobacco and alcohol use. Subgroup differences were assessed using adjusted logistic regression models with interaction terms; estimated predictive margins were graphed for interpretability.

Data were weighted to represent the U.S. general population, adjusting for survey design, sampling, probability of selection (based on household size), and survey mode. For the current analysis, survey weights were averaged, so they represent the estimated population at the midpoint between the two surveys. Sensitivity analyses tested interactions of survey dataset with key predictor variables in adjusted models. All analyses were conducted using Stata version 16.1 [34].

## Results

Demographic and substance use differences between respondents in alcohol control states and the other states are shown in Table 1. Other than race and ethnicity (higher proportion White residents and lower proportion Hispanic residents in control states), there were no statistically significant demographic differences between respondents in control states and the other states. There was no statistically significant difference in lifetime tobacco use status, but there was a significant difference in daily tobacco use status in control states compared to the other states (control states had a lower proportion of people who never used tobacco daily and a higher proportion of people who currently use tobacco daily). Control states had lower proportions of people who currently drink and higher proportions of both people who used to drink and lifetime abstainers who never drank alcohol. Control states also had significantly lower proportions of people who drank wine and people who drank spirits (including drinking spirits monthly) in the past year, but no statistically significant difference in the proportion of people who drank beer. When analyses were limited to people who consumed alcohol in the past year, there remained significant differences for past-year wine drinking (69.8% vs. 73.9%, Design-based *F*(1, 14,794) = 5.56, *p* = 0.02) and monthly spirits drinking (37.3% vs. 42.6%, Design-based *F*(1, 14,784) = 7.82, *p* = 0.005) in control states. There were no statistically significant differences in AUD status or recovery status for people in control states compared to people in the other states.

**Table 1:** Characteristics of Respondents in Control States and Other States, 2015 and 2020 National Alcohol Surveys (N = 14,817)

	Control States		Other States		Total
	%	95% CI	%	95% CI	%
Gender/Sex of Respondent					
Female (N = 9,305)	52.7	(49.9,55.5)	51.6	(50.3,52.9)	51.8
Design-based F(1, 15,650) = 0.5274; P = 0.468					
Race or Ethnicity					
White (N = 7,801)	74.7	(72.3,76.9)	62.4	(61.3,63.6)	64.7
Black (N = 3,500)	12.2	(10.7,13.8)	12.6	(11.9,13.3)	12.5
Hispanic (N = 3,198)	7.1	(5.9,8.7)	16.6	(15.8,17.5)	14.9
Design-based F(2.94, 45970.95) = 34.9397; P < 0.001					
Education					
High School or Less (N = 4,784)	35.3	(32.6,38.0)	35.2	(34.0,36.5)	35.2
Design-based F(1, 15,616) = 0; P = 0.995					
Tobacco Use Status					
Never Used Tobacco (N = 9,894)	58.3	(55.6,61.0)	60.3	(59.0,61.5)	59.9
Prior Tobacco Use (N = 2,945)	19.1	(17.1,21.3)	19.8	(18.8,20.8)	19.6
Current Tobacco Use (N = 2,692)	22.6	(20.2,25.1)	20	(18.9,21.1)	20.4
Design-based F(1.99, 30842.09) = 2.0807; P = 0.125					
Daily Tobacco Use Status					
Never Used Tobacco Daily (N = 10,926)	63.6	(60.9,66.3)	67.3	(66.0,68.5)	66.6
Prior Daily Tobacco Use (N = 2,432)	17.4	(15.4,19.6)	17	(16.1,18.0)	17.1
Current Daily Tobacco Use (N = 2,033)	19	(16.8,21.4)	15.7	(14.8,16.7)	16.3
Design-based F(1.99, 30590.31) = 4.1224; P = 0.016					
Drinking Status					
Currently Drinks (N = 10,550)	66	(63.4,68.6)	70.7	(69.5,71.8)	69.8
Used to Drink (N = 3,037)	20.6	(18.4,22.9)	18.7	(17.7,19.7)	19
Lifetime Abstainer (N = 2,072)	13.4	(11.8,15.3)	10.6	(9.9,11.4)	11.2
Design-based F(1.99, 31145.73) = 6.4659; P = 0.002					
Drank Beer Past Year					
Yes (N = 6,705)	45.2	(42.5,48.0)	47.6	(46.3,48.8)	47.1
Design-based F(1, 15,576) = 2.2558; P = 0.133					
Drank Wine Past Year					
Yes (N = 7,888)	45.8	(43.1,48.6)	51.9	(50.6,53.1)	50.8
Design-based F(1, 15,585) = 15.3172; P < 0.001					
Drank Spirits Past Year					
Yes (N = 6,852)	44.1	(41.4,46.9)	47.3	(46.0,48.5)	46.7
Design-based F(1, 15,589) = 4.0393; P = 0.044					
Drank Spirits At Least Monthly Past Year					
Yes (N = 4,231)	24.5	(22.2,27.0)	30.1	(28.9,31.3)	29.1
Design-based F(1, 15,589) = 15.5467; P < 0.001					
AUD Status					
Never 2 + AUD Symptoms (N = 11,240)	68.8	(66.1,71.4)	68.3	(67.1,69.5)	68.4
Prior 2 + AUD Symptoms (N = 3,133)	23.9	(21.5,26.5)	22.5	(21.4,23.6)	22.8
Current 2 + AUD Symptoms (N = 1,227)	7.3	(5.9,8.9)	9.2	(8.5,10.0)	8.9
Design-based F(2, 31180.53) = 2.4035; P = 0.09					
In Recovery					
Yes (N = 959)	6.1	(4.9,7.6)	7	(6.4,7.7)	6.9
Design-based F(1, 15,551) = 1.2461; P = 0.264					

Note. Limited to respondents ages 21 and older. Totals may not sum to 100% (not all groups shown ).

### What proportion of people who smoke daily may be at risk of relapse to problem drinking if they could only purchase tobacco in liquor stores?

In the full weighted sample (representing 221 million U.S. adults ages 21 and older), there was a significant association of past-year drinking and tobacco use (Design-based *F*(1.98, 29187.41) = 49.40, *p* < 0.001), with 23.0% of people who drink currently and 21.5% of people who used to drink, but only 6.7% of lifetime alcohol abstainers, reporting past-year tobacco use. Among people who used tobacco, most (76.8%) consumed alcohol in the past year, but one-fifth (20.0%) used to drink alcohol but did not do so in the past year. The association between daily smoking and drinking status also was significant (Design-based *F*(1.98, 29072.88) = 37.20, *p* < 0.001), with 17.6% of people who drank in the past year and 19.2% of people who used to drink, but only 5.3% of lifetime alcohol abstainers, reporting daily smoking. Daily smoking also was significantly associated with AUD status (Design-based *F*(1.99, 29230.58) = 117.66, *p* < 0.001), with 31.7% of people with current AUD and 26.1% with prior AUD, but only 11.6% with no AUD history, reporting daily smoking. Among people who smoke daily, 16.2% had current AUD and another 36.5% had prior AUD. The association between daily smoking and recovery status also was significant (Design-based *F*(1, 14,608) = 332.72, *p* < 0.001), with 49.0% of people in recovery, but only 14.3% of people who are not in recovery, reporting daily smoking. These associations were not significantly different in control states compared to the other states.

In the subset of respondents from control states (representing 41.85 million U.S. adults ages 21 and older), there also was a significant association of past-year drinking and tobacco use (Design-based *F*(1.98, 29361.71) = 16.46, *p* < 0.001), with most people who use tobacco (76.3%) reporting past-year drinking, and one-fifth (20.8% or 2.01 million adults) reporting prior drinking. With increased exposure to alcohol cues in the retail environment, these 2 million people in control states who use tobacco and who used to drink, but no longer do so, could be at risk of relapse to drinking if they were required to purchase tobacco in liquor stores. As in the full sample, the association between daily smoking and drinking status was statistically significant (Design-based *F*(1.95, 28938.71) = 13.70, *p* < 0.001), with most people who smoke daily (74.1%) reporting past-year drinking, and almost one-quarter (23.3% or 1.89 million adults) reporting prior drinking (only 2.6% of people who smoke daily were lifetime abstainers). Daily smoking also was significantly associated with AUD status for respondents in control states (Design-based *F*(2, 29559.57) = 32.09, *p* < 0.001), with 11.1% of people who smoke daily reporting current AUD (901,000 adults) and another 44.0% of people who smoke daily (3.56 million adults) reporting prior AUD. The association between daily smoking and recovery status also was statistically significant (Design-based *F*(1, 14,787) = 78.84, *p* < 0.001), with 58.8% of people in recovery (1.50 million adults), but only 17.0% of people who are not in recovery (an additional 6.60 million adults), reporting daily smoking. With increased exposure to retail alcohol purchasing cues, these 3.56 million people with a history of AUD and 1.5 million adults in recovery who smoke daily could be at risk of relapse to problem drinking under this hypothetical scenario for shifting tobacco retail policy in alcohol control states.

### What proportion of people who smoke daily and who also drink alcohol would be at risk of purchasing higher-strength ABV if they could only purchase tobacco in liquor stores?

In the full sample of people who consumed alcohol in the past year, there was not a significant association of spirits drinking with daily tobacco use (Design-based *F*(1, 14,695) = 0.80, *p* > 0.10), with 17.2% of people who drink spirits and 18.3% of people who do not drink spirits reporting daily tobacco use. Among people who used tobacco daily and who also drank alcohol in the past year, a majority (65.8%) drank spirits. This association was not significantly different in control states compared to the other states. However, limiting to respondents in control states, there was a significant association of spirits drinking and lifetime daily tobacco use (Design-based *F*(1.99, 29411.48) = 4.07, *p* = 0.02), with about half (48.5%) of people who currently smoke daily and 51.2% of people who used to smoke daily also reporting drinking spirits (compared to 41.2% of people who never smoked daily). This proposed policy shift potentially would increase exposure to strong spirits for an estimated 4.11 million people in control states who smoke daily but do not currently drink spirits. We also could consider risk of relapse to smoking by people who drink spirits: 20.5% of people who drank spirits in the past year (3.80 million adults in control states) used to smoke daily, and they may be at increased risk of relapse to smoking if they were exposed to tobacco products in liquor stores.

### Are these patterns different for high-priority subgroups?

**Fig. 1:**
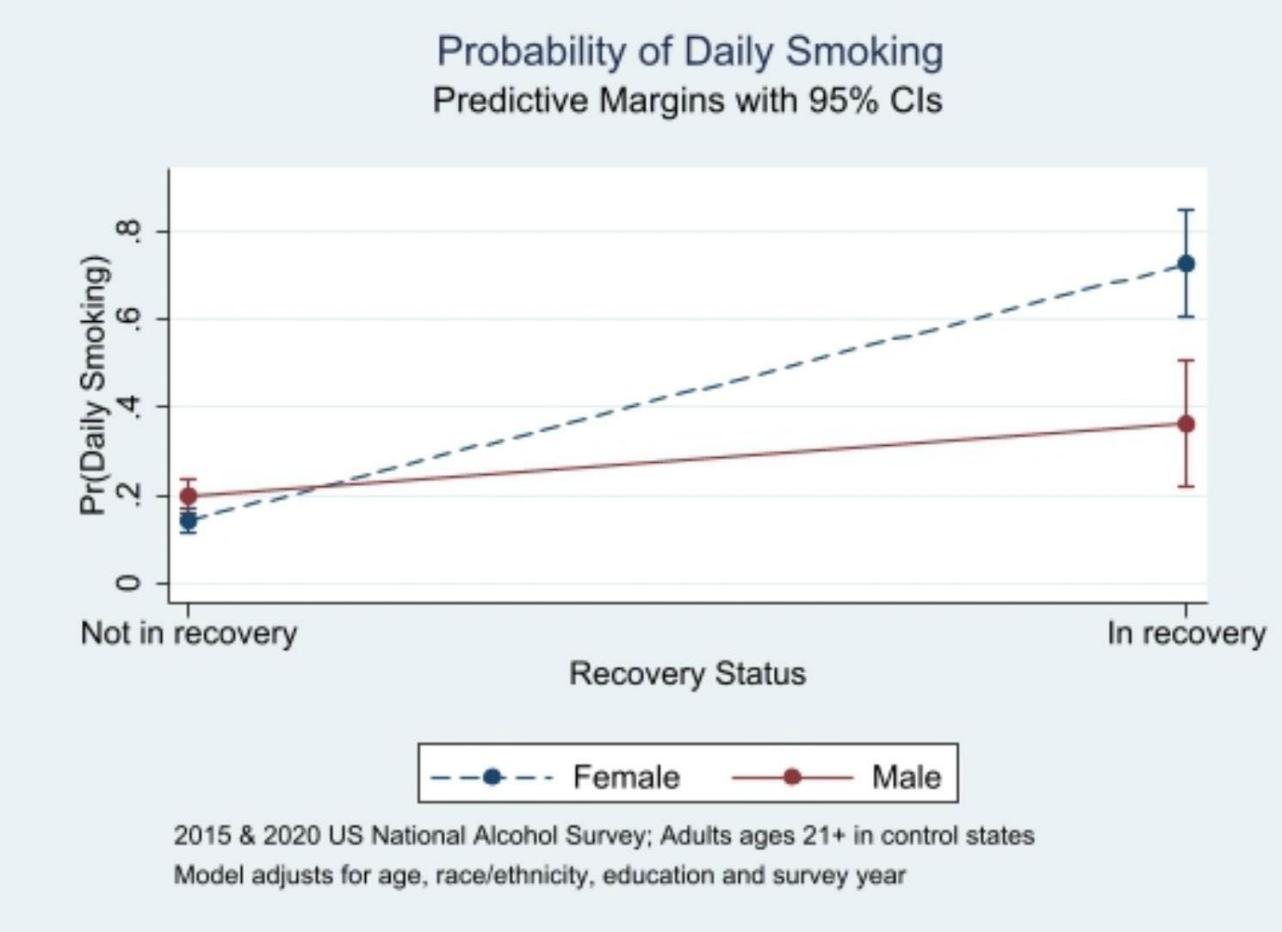
Gender/Sex Differences in Daily Smoking by Recovery Status

Contrary to expectations, none of the associations varied significantly by respondent race or ethnicity. Only one relationship varied significantly by gender: In an adjusted logistic regression model, there was a significant interaction of gender with recovery status in relation to daily smoking (Table 2). This association was stronger for women than for men (Fig. 1), with the average marginal effect of recovery status on daily smoking being 0.586 (95% CI = 0.461–0.710; *p* < 0.001) for women and 0.162 (95% CI = 0.014–0.311; *p* = 0.03) for men. Unadjusted population estimates translate to an estimated 951,000 women in recovery from an alcohol and/or drug problem who smoke daily (76.2% of women in recovery and 4.3% of all women in control states), and an additional estimated 546,000 men in recovery who smoke daily (42.1% of men in recovery and 2.8% of all men in control states). These people in recovery who smoke daily, particularly women, may be at risk of relapse to AUD if exposed to increased alcohol cues when purchasing tobacco.

**Table 2:** Subgroup Differences in Associations of Recovery Status and Lifetime AUD with Daily Smoking for Respondents in Control States, 2015 and 2020 National Alcohol Surveys (n = 2,245)

Model 1	OR	(95% CI)	p-value
In Recovery	19.99	(10.03,39.83)	< 0.001
Male	1.54	(1.10,2.15)	0.01
Recovery X Male	0.12	(0.04,0.33)	< 0.001
Some college or more (vs. high school or less)	0.39	(0.28,0.55)	< 0.001
Age	0.99	(0.98,0.99)	0.001
Black (vs. Non-Hispanic White)	0.98	(0.66,1.47)	0.94
Hispanic (vs. Non-Hispanic White)	0.81	(0.43,1.53)	0.52
Other Race or ethnicity (vs. Non-Hispanic White)	0.78	(0.39,1.54)	0.47
2020 NAS (vs. 2015 NAS)	0.53	(0.39,0.72)	< 0.001
Model 2	OR	(95% CI)	p-value
Lifetime AUD	5.14	(3.08,8.58)	< 0.001
Male	1.08	(0.78,1.51)	0.63
Some college or more (vs. high school or less)	0.49	(0.32,0.75)	0.001
AUD X Education	0.50	(0.26,0.96)	0.04
Age	0.99	(0.98,1.00)	0.03
Black (vs. Non-Hispanic White)	1.10	(0.75,1.61)	0.64
Hispanic (vs. Non-Hispanic White)	0.77	(0.42,1.43)	0.41
Other Race/ethnicity (vs. Non-Hispanic White)	0.97	(0.50,1.90)	0.94
2020 NAS (vs. 2015 NAS)	0.60	(0.44,0.83)	0.002

**Fig. 2:**
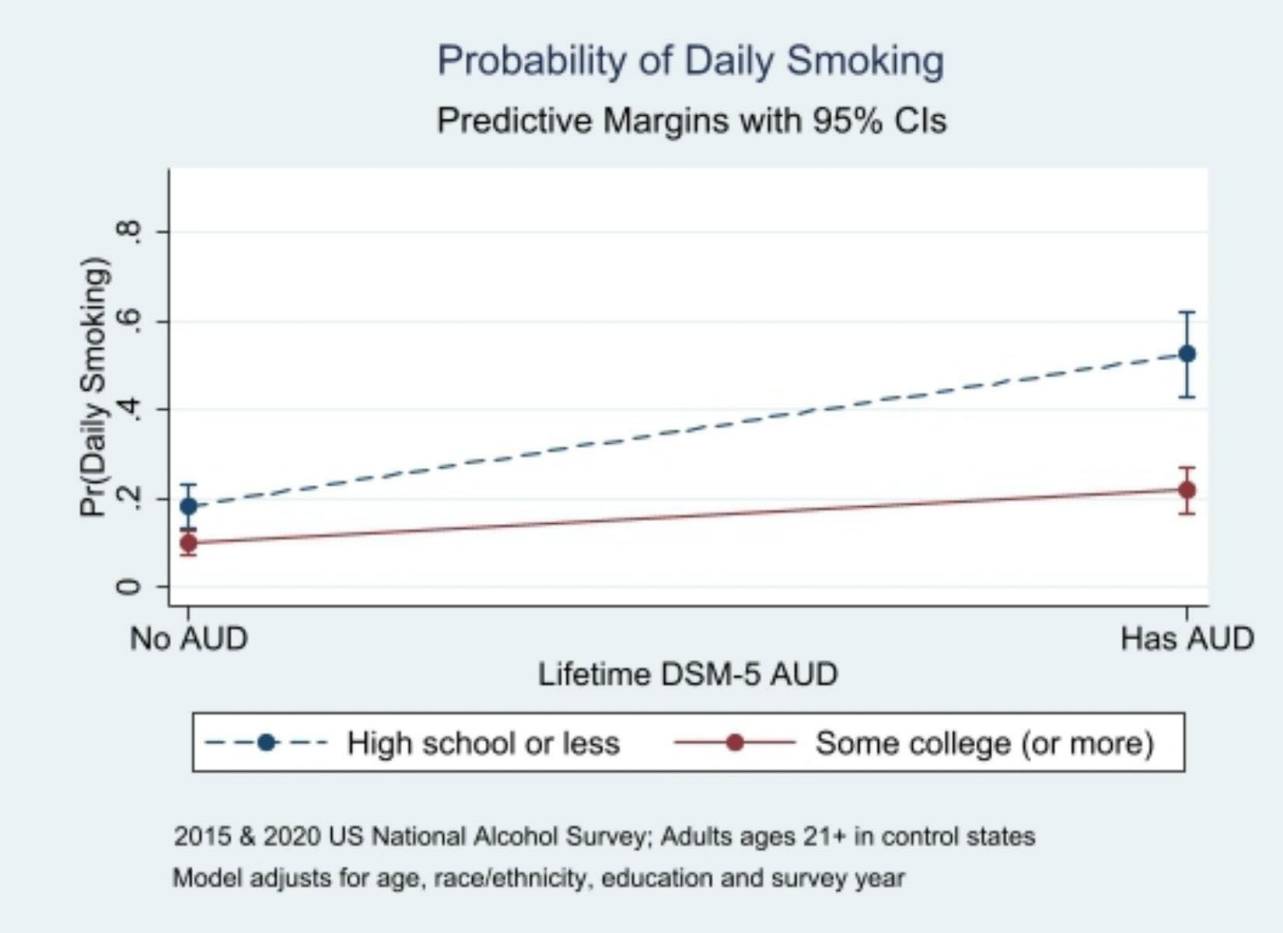
Education Differences in Daily Smoking by Lifetime AUD Status

Similarly, another relationship varied significantly by education: In an adjusted logistic regression model, there was a significant interaction of education with lifetime AUD status (dichotomized as ever vs. never) in relation to daily smoking (Table 2). This association was stronger for people with lower levels of education than for those with higher levels of education (Fig. 2), with the average marginal effect of lifetime AUD on daily smoking being 0.343 (95% CI = 0.235–0.451; *p* < 0.001) for those with low education and 0.119 (95% CI = 0.059–0.178; *p* < 0.001) for those with high education. Unadjusted population estimates from control states translate to an estimated 2.37 million people with low education who have lifetime AUD and also smoke daily (55.1% daily smoking among those with lifetime AUD and 16.7% overall, in people with low education), and an additional estimated 2.09 million people with higher education who have lifetime AUD and also smoke daily (23.8% daily smoking among those with lifetime AUD and 7.7% overall, in people with high education). These people with lower levels of education who smoke daily and have a history of AUD may be at higher risk of relapse to AUD if tobacco retail policy changes were to increase their exposure to alcohol cues.

Sensitivity analyses showed that recovery status and lifetime AUD status had strong associations with daily smoking among adults ages 21 or older in control states, and there were no statistically significant interactions of either recovery status or lifetime AUD with dataset (both *p* > 0.10), suggesting these relationships with daily smoking were not markedly different from one survey to the next. There also was a protective relationship of higher education with daily smoking that did not interact with dataset either (*p* > 0.10). However, the association of gender with daily smoking did vary significantly across datasets (*p* < 0.05), with the gender differences seen in the earlier survey being less pronounced in the later survey. Specifically, predictive margins showed the probability of daily smoking for women ages 21 or older in control states went from 19.9% (95% CI = 15.7–24.1) in 2015 to 16.2% (95% CI = 13.0-19.4) in 2020, but the probability for men went from 28.9% (95% CI = 22.6–35.2) to 13.6% (95% CI = 9.8–17.4) over the same period.

## Discussion

Given recent U.S. national tobacco policy changes increasing the federal minimum legal sales age for tobacco to 21 years—the same as for all alcohol products—we examined whether there might be potential unintended negative consequences of a hypothetical tobacco endgame strategy [5, 35] to move all retail sales for combustible tobacco to state liquor stores in states with a retail monopoly system. This descriptive analysis of two large national survey datasets showed strong associations between alcohol and tobacco use overall and in a subsample of respondents from the states with government control over retail sale of spirits. Further, some relationships between indicators of alcohol problems and daily smoking were stronger for women than men and for people with lower levels of education than those with more education. While these findings regarding co-use of alcohol and tobacco are similar to those from prior national epidemiologic data collected almost two decades ago [20], the pattern of results suggests there could be negative impacts of potential tobacco retail changes, including risk for relapse to drinking among people who smoke daily who also have a history of alcohol problems, especially for women and people with low education who are living in alcohol control states. Exposure to state liquor stores that sell tobacco could place people who smoke and who also have a history of AUD at particularly elevated risk, as they are a group that is especially vulnerable to alcohol marketing due to increased salience of alcohol-related cues [36].

Only about half of people who smoked daily in the control state sample reported that they drank spirits in the past year, which implies that there also could be a risk of some of these people who smoke changing their alcohol consumption to include spirits if they were repeatedly exposed to a state-run liquor store retail environment to purchase tobacco products. Additionally, given patterns of bulk tobacco purchasing (i.e., buying cigarettes by the carton), by some estimates, perhaps one quarter to one third [29] of adults living in alcohol control states who smoke daily and drink alcohol—which translates to about 2 million adults—also may be motivated by cost-savings of buying alcohol in large volumes; however, we did not have data on purchasing to allow analysis of this important question. Further, people who drink but who have stopped smoking may start smoking again if tobacco is present in state alcohol stores, as evidence suggests exposure to tobacco marketing is associated with failed quit attempts [37, 38] as well as relapse to smoking [39, 40], and purchases of tobacco and alcohol have been found to cluster together [41].

Another concern is that people who smoke who also buy liquor may upgrade to purchase larger bottles when they buy cigarettes at stores that sell larger volumes at lower prices, but we were not able to address this issue in these data. Currently, in all U.S. states, people can buy beer and/or wine at stores that also sell tobacco products. However, many tobacco retail outlets also offer other products in addition to alcohol, including gasoline, food, and pharmacy items, thereby making the triggering effect of alcohol cues less concentrated than in a retail environment dedicated to alcohol sales. People in recovery from alcohol problems who smoke, as well as people who drink who used to smoke, might be affected differently by a retail environment dedicated only to alcohol and tobacco. We did not detect any statistically significant differences in the associations between alcohol and tobacco use, including the relationship between daily smoking and past-year spirits drinking, in control states compared to the other states, however. This suggests that other factors, such as common biobehavioral predispositions [21, 22], may be more important for co-use than the retail context.

Although there may be some potential drawbacks to limiting tobacco retail sales to state-run liquor stores, there also are many possible benefits. Reducing overall tobacco availability would likely reduce smoking prevalence [12, 13], particularly youth smoking [14–16] and tobacco use initiation [17], beyond the effects of Tobacco 21 legislation, which already has been shown to decrease young adult use in states that adopted the higher sales age prior to institution of the federal law [42, 43]. There also may be reductions in over-exposure to tobacco retail in historically socially and economically disadvantaged neighborhoods [44–47], which could reduce tobacco-related disparities in morbidity and mortality in states that adopt this approach to tobacco sales regulation. Further, reductions in tobacco retail density also may prevent smoking relapse and help people successfully quit smoking [48, 49]. Finally, legislators may view this method of reducing tobacco availability as an acceptably “incremental” change, less dramatic than some other proposals to end retail sales completely [50]. However, to date, no U.S. state governments, nor any other known federal governments, have implemented a government retail control model for tobacco in which sales are restricted to state-owned or state-contracted stores. Some countries such as Hungary [51], France [52] and Spain [53] restrict tobacco sales to specific types of stores. In Spain, these stores only sell tobacco products (they do not sell alcohol) [53], but in Hungary they also are allowed to sell alcohol [51]. Comparative research would be valuable for understanding how national and local contexts may impact alcohol and tobacco purchasing and consumption patterns.

Depending on how a control system decided to set up tobacco sales under this hypothetical tobacco endgame strategy [5, 35] to move retail sales for combustible tobacco to state liquor stores, there could be policy protections put in place, such as restrictions on tobacco point-of-sale advertising and self-service [54] as well as state-determined tobacco minimum pricing and elimination of discounts and coupons [55, 56]. Some of these control states (e.g., Utah) already have some of these policy protections for alcohol, including no exterior advertising and no manufacturer discounts or coupons, so extending those policies to cover tobacco products would presumably be feasible. Further, tobacco products could be stored out of sight (behind a counter and in closed cabinets), and there also could be physical barriers between tobacco and alcohol sales areas (including separate entrances) to reduce exposure of targeted consumers to the other product. Some control states (e.g., Alabama) already require separate entrances (and separate sales areas) for state liquor stores that are adjacent to convenience stores that sell other products, which suggest that this structural solution also may be feasible. The retail sector is the focus of many endgame approaches, and transitioning tobacco sales to state run liquor stores would be in keeping with approaches that try to reduce the number of retail outlets (such as ending tobacco sales in pharmacies or putting caps on the number of retailers granted licenses).

### Study Strengths and Limitations

The large samples in these U.S. national datasets permitted examination of key subgroups of interest, including groups based on self-reported gender, level of education, race and ethnicity. Despite the large sample size, we were not able to assess differences in alcohol consumption by the type of state alcohol control system. Future work designed to test pre-registered hypotheses in this area would be informative to verify the findings from this exploratory, descriptive study and to overcome the key limitation that these survey data were not collected specifically to address the research question at hand. Another limitation pertains to the cross-sectional data. Studies using longitudinal data are needed to better understand relationships between alcohol and tobacco consumption over time, particularly in relation to subgroup differences in co-use. Co-use patterns also may be changing along with demographic shifts in smoking, as suggested by the narrowing of gender differences in daily smoking in the 2020 survey data analyzed here. Additional insights also may be gained from longitudinal administrative data or retail sales data on purchases of alcohol and tobacco products (see, for example [41] for a study using grocery store scanner data in Finland and [57] for a study of alcohol and tobacco purchasing in the United Kingdom), although these types of data likely would not contain information on the purchasers’ behavioral health history or demographic characteristics that is helpful for understanding groups that may be at elevated risk. Thus, as tobacco and alcohol policies continue to evolve at the national, state and local levels, ongoing epidemiological surveillance of consumer behavior (purchasing patterns including place and quantity of alcohol and tobacco purchases), drinking behavior (particularly heavy drinking and consumption of high ABV beverages), and smoking practices (including usual number of cigarettes per day and quit attempts) will be important for understanding synergies between these different policy dimensions.

## Conclusion

There could be negative consequences of an endgame strategy to restructure tobacco retail sales by limiting purchase locations to state-run liquor stores. These potential consequences may include increased risk for relapse to drinking among people with a history of alcohol problems who smoke daily, especially among women and people with low levels of education. Given the enormous toll of tobacco on health and health equity across the U.S., in particular for some marginalized groups, thoughtful policy decisions should be taken to supplement the potential benefits of tobacco retail policy changes with procedures to mitigate unintended harms.

## Data Availability

Datasets from the NAS series through 2010 are publicly available from https://arg.org/nas-datasets/. The 2015 and 2020 NAS datasets are available upon request under terms of a data use agreement with the Public Health Institute’s Alcohol Research Group.
